# Oxidative Stress-Induced Afterdepolarizations and Protein Kinase C Signaling

**DOI:** 10.3390/ijms18040688

**Published:** 2017-03-30

**Authors:** Yu-Dong Fei, Wei Li, Jian-Wen Hou, Kai Guo, Xiao-Meng Chen, Yi-He Chen, Qian Wang, Xiao-Lei Xu, Yue-Peng Wang, Yi-Gang Li

**Affiliations:** 1 Department of Cardiology, Xin Hua Hospital Affiliated to Shanghai Jiao Tong University School of Medicine, Shanghai 200092, China; fyd6254@gmail.com (Y.-D.F.); lwcbss@163.com (W.L.); houjianwena@126.com (J.-W.H.); guokai0201@aliyun.com (K.G.); chenxmcorrine@outlook.com (X.-M.C.); cyh1726@163.com (Y.-H.C.); wangq0327@163.com (Q.W.); 2 Department of Biochemistry and Molecular Biology, Division of Cardiovascular Diseases, Mayo Clinic, 200 First St SW, Rochester, MN 55905, USA; xu.xiaolei@mayo.edu

**Keywords:** oxidative stress, afterdepolarization, triggered activity, protein kinase C, arrhythmia

## Abstract

Background: Hydrogen peroxide (H_2_O_2_)-induced oxidative stress has been demonstrated to induce afterdepolarizations and triggered activities in isolated myocytes, but the underlying mechanisms remain not fully understood. We aimed to explore whether protein kinase C (PKC) activation plays an important role in oxidative stress-induced afterdepolarizations. Methods: Action potentials and ion currents of isolated rabbit cardiomyocytes were recorded using the patch clamp technique. H_2_O_2_ (1 mM) was perfused to induce oxidative stress and the specific classical PKC inhibitor, Gö 6983 (1 μM), was applied to test the involvement of PKC. Results: H_2_O_2_ perfusion prolonged the action potential duration and induced afterdepolarizations. Pretreatment with Gö 6983 prevented the emergence of H_2_O_2_-induced afterdepolarizations. Additional application of Gö 6983 with H_2_O_2_ effectively suppressed H_2_O_2_-induced afterdepolarizations. H_2_O_2_ increased the late sodium current (I_Na,L_) (*n* = 7, *p* < 0.01) and the L-type calcium current (I_Ca,L_) (*n* = 5, *p* < 0.01), which were significantly reversed by Gö 6983 (*p* < 0.01). H_2_O_2_ also increased the transient outward potassium current (I_to_) (*n* = 6, *p* < 0.05). However, Gö 6983 showed little effect on H_2_O_2_-induced enhancement of I_to_. Conclusions: H_2_O_2_ induced afterdepolarizations via the activation of PKC and the enhancement of I_Ca,L_ and I_Na,L_. These results provide evidence of a link between oxidative stress, PKC activation and afterdepolarizations.

## 1. Introduction

Reactive oxygen species (ROS) play different roles in physiological and pathological conditions. Under pathological conditions, such as heart failure (HF) [1] and ischemia/reperfusion [2], elevated ROS levels can predispose the heart to arrhythmias. Previous studies evidenced hydrogen peroxide (H_2_O_2_)-induced oxidative stress [3] and it is known that H_2_O_2_ can affect various ion channels, such as the L-type calcium currents (I_Ca,L_) [4,5], transient outward potassium currents (I_to_) [6,7], late sodium currents (I_Na,L_) [8,9], and further induce electrical abnormalities including early afterdepolarizations (EADs), delayed afterdepolarizations (DADs) and triggered activities (TAs) [3,8].

Protein kinase C (PKC), a group of serine/threonine protein kinase enzymes, has been identified as an important regulator of a variety of cardiac responses including myocardial fibrosis, cardiomyocyte hypertrophy and inflammation, which are closely associated with heart failure and ischemia [10,11,12]. PKC levels have been found to be significantly elevated in both HF animal models and in patients with HF [13,14], and cardioprotective effects were observed by selective inhibition of PKC isozymes [11,15]. Moreover, PKC is acknowledged to be closely associated with various ion channels and transporters [5]. PKC activation has been shown to facilitate the induction of ventricular fibrillation, which can be reversed by PKC inhibition [16].

Accumulating evidence suggests that increased oxidative stress could serve as an important arrhythmogemic factor in diseased hearts. ROS can persistently activate PKC via direct oxidative modification [17] and increasing the cellular level of inositol-1,4,5-trisphosphate and diacylglycerol [18]. Up until now, there was only scant report on the role of PKC in oxidative stress-induced afterdepolarizations and its ionic mechanisms. In the present study, we tested the hypothesis that oxidative stress may activate PKC and enhance arrhythmogenic activity in cardiomyocytes, and focused on the following issues: (1) whether PKC signaling was involved in oxidative stress-induced afterdepolarizations; (2) the underlying ionic mechanisms. Our study showed that PKC activation plays an important role in oxidative stress-induced afterdepolarizations, and augmentation of I_Ca,L_ and I_Na,L_ seems to be the underlying mechanism.

## 2. Results

### 2.1. H_2_O_2_-Induced Afterdepolarizations in Adult Rabbit Ventricular Myocytes

Action potentials (APs) were recorded from isolated rabbit ventricular myocytes using the whole-cell current clamp mode. To elicit EADs stably, the myocytes were paced at basic cycle lengths of 6 s [3]. After the action potential duration (APD) and morphology reached steady state, cells were perfused with bath solution containing 1 mM H_2_O_2_.

**Figure 1:**
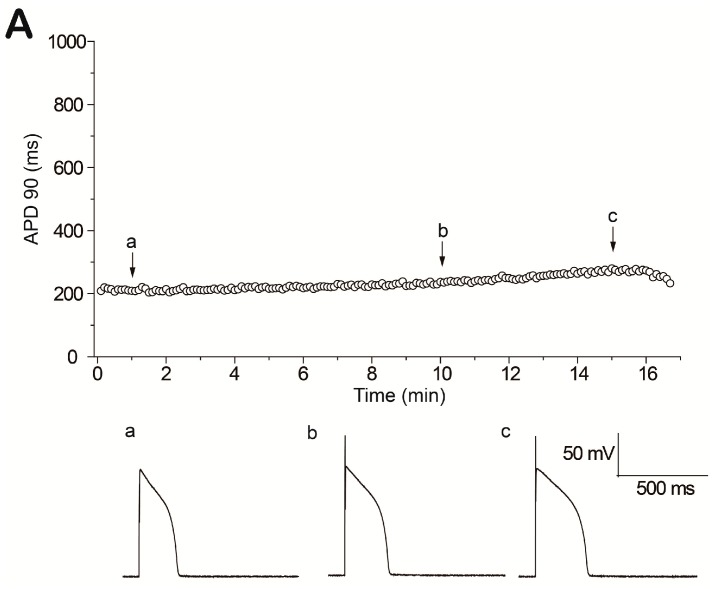

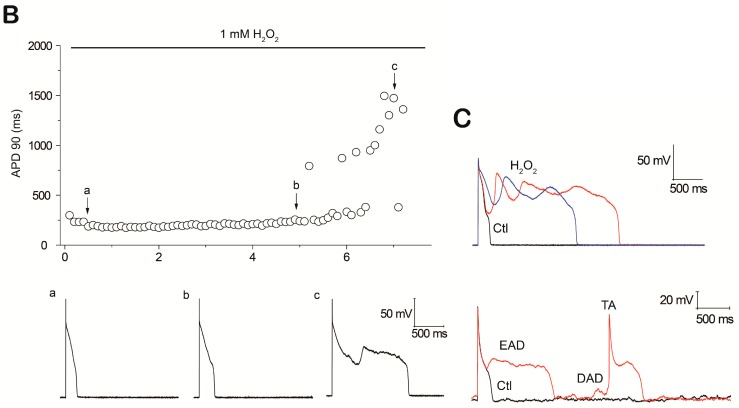
Afterdepolarizations induced by H_2_O_2_ perfusion. (**A**) Action potentials (APs) were elicited consecutively at basic cycle lengths of 6 s and values of action potential durations (APD) 90 were plotted over time. APD 90 was consecutively recorded from a cell perfused with standard Tyrode solution for over 15 min. APs at 1 min (a), 10 min (b), and 15 min (c) are shown below. No early afterdepolarizations (EADs), delayed afterdepolarizations (DADs) or triggered activities (TAs) occurred; (**B**) H_2_O_2_ (1 mM) was perfused continuously as indicated by the horizontal bar. APs at the beginning of the perfusion (a), and after perfusion with H_2_O_2_ for 5 min (b) and 7 min (c) are shown below; (**C**) Examples of afterdepolarizations and TAs during H_2_O_2_ exposure, including multiple oscillatory EADs (above), and different electrical abnormalities in a pacing cycle (below).

As shown in Figure 1, H_2_O_2_ perfusion prolonged APD gradually and induced afterdepolarizations and TAs after approximately 7 min, while control experiments in parallel (without H_2_O_2_) showed that no electrical abnormalities occurred for up to 15 min (*n* = 9). APD 90 was prolonged from 276.7 ± 77.4 to 585.0 ± 65.0 ms (*n* = 9, *p* < 0.01) at 5 min of application of 1 mM H_2_O_2_. Examples of afterdepolarizations and TAs are shown in Figure 1C.

### 2.2. The Role of PKC Signaling in H_2_O_2_-Induced Afterdepolarizations

**Figure 2:**
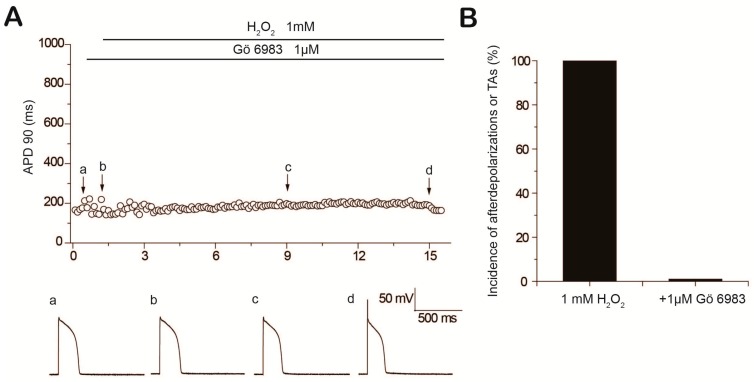
Prevention of H_2_O_2_-induced early afterdepolarizations (EADs) by the protein kinase C inhibitor Gö 6983. (**A**) Time course of action potential duration (APD) 90 in a myocyte treated with Gö 6983 before exposure to 1 mM H_2_O_2_. Action potentials under control conditions (a), in the presence of Gö 6983 (b); after perfusion of H_2_O_2_ for 8 min (c) and 14 min (d) are shown below; (**B**) Incidence of EADs, delayed afterdepolarizations (DADs) or triggered activities (TAs) in the presence of H_2_O_2_ and pretreated with Gö 6983.

Next we tested whether PKC activation was involved in H_2_O_2_-induced afterdepolarizations by using the specific classical PKC inhibitor Gö 6983. Unlike those myocytes consistently presenting EADs approximately 7 min after exposure to 1 mM H_2_O_2_ (Figure 1A), pretreatment with Gö 6983 (1 μM) prevented the emergence of H_2_O_2_-induced EADs for up to 15 min (Figure 2A). As shown in Figure 2B, the incidence of EADs induced by H_2_O_2_ was significantly reduced by pretreatment with Gö 6983 (100% vs. 0%, *n* = 8). To further confirm the effect of PKC inhibition on H_2_O_2_-induced EADs, we applied another widely used selective PKC inhibitor, Bisindolylmaleimide (BIM). As expected, pretreatment with BIM (1 μM) prevented the emergence of H_2_O_2_-induced afterdepolarizations in six of six ventricular myocytes (Figure S1A).

**Figure 3:**
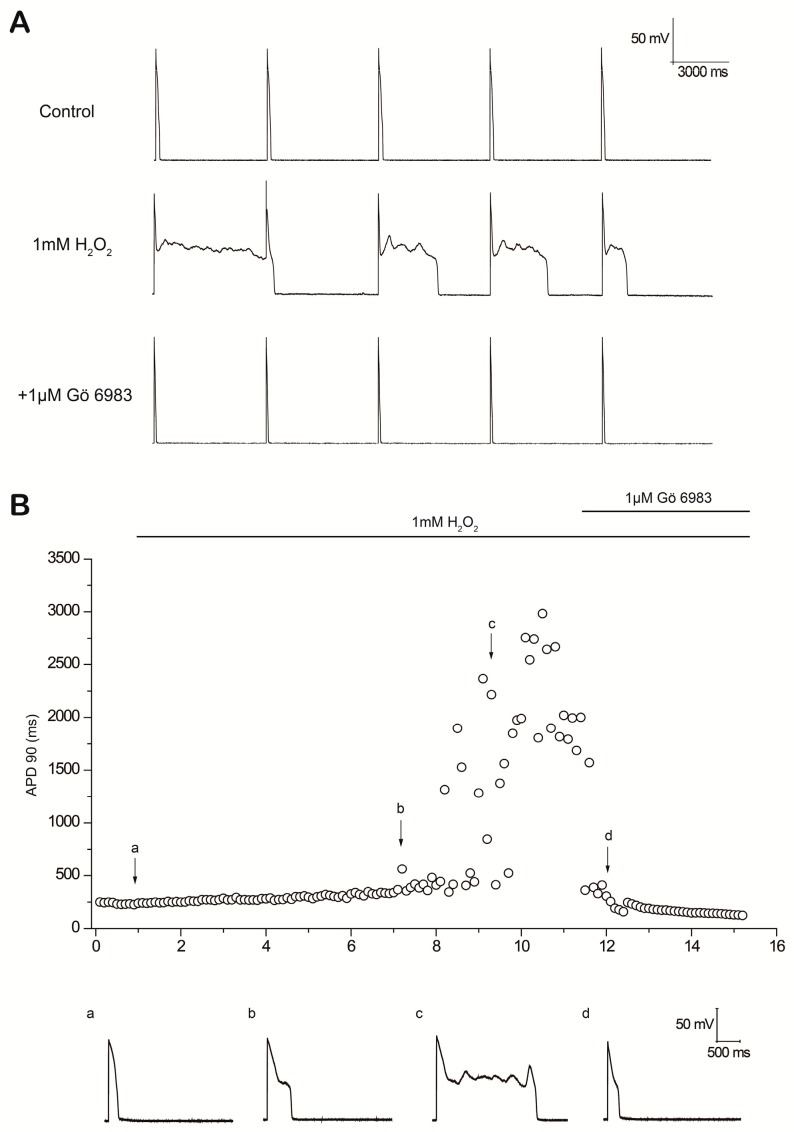
Suppression of H_2_O_2_-induced early afterdepolarizations (EADs) by the PKC inhibitor Gö 6983. (**A**) Gö 6983 completely suppressed all H_2_O_2_-induced EADs and significantly shortened action potential duration (APD). The representative five consecutive action potentials (APs) are shown in each period; (**B**) Time course of APD 90 in a myocyte treated with Gö 6983 after EADs were induced by H_2_O_2_. APs under control conditions (a), after perfusion with H_2_O_2_ for 6 min (b) and 8 min (c), and after application of Gö 6983 (d) are shown below.

In another series of experiments, after EADs were induced by H_2_O_2_ perfusion, myocytes were perfused with bath solution containing both Gö 6983 and H_2_O_2_. Gö 6983 effectively suppressed H_2_O_2_-induced EADs, DADs and TAs in five out of five myocytes. Five consecutive APs under control conditions, in the presence of H_2_O_2_ and after the addition of Gö 6983, are shown in Figure 3A. Values of APD 90 are plotted over time in Figure 3B. In another group of myocytes, BIM was applied after EADs were induced by H_2_O_2_ perfusion, and BIM also effectively reversed EADs in five of five myocytes (Figure S1B).

### 2.3. PKC Mediates I_Ca,L_ Enhancement in H_2_O_2_-Induced Afterdepolarizations

**Figure 4:**
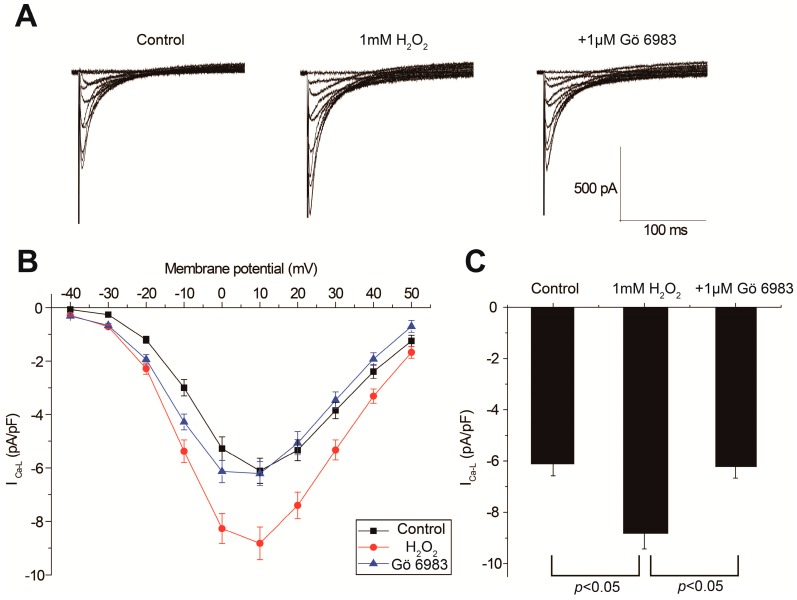
Effects of Gö 6983 on H_2_O_2_-induced L-type calcium current (I_Ca,L_) elevation. Representative current-voltage traces (**A**) and the averaged current-voltage curves (**B**) showed that H_2_O_2_ significantly augmented I_Ca,L_, which was reversed by Gö 6983. Summary histogram (**C**) of I_Ca,L_ at +10 mV under control condition, in the presence of 1 mM H_2_O_2_ alone or plus 1 μM Gö 6983.

The effects of H_2_O_2_ and the PKC inhibitor on the major membrane currents were analyzed using a voltage clamp. The representative current-voltage traces of I_Ca,L_ are displayed in Figure 4A, and the averaged I–V curves (*n* = 5) are shown in Figure 4B. Our results show that 1 mM H_2_O_2_ significantly enhanced I_Ca,L_, which was effectively attenuated by Gö 6983. The peak amplitudes of I_Ca,L_ at +10 mV were measured for data analysis. As shown in Figure 4C, I_Ca,L_ was elevated from −6.11 ± 0.47 to −8.82 ± 0.61 pA/pF after H_2_O_2_ perfusion (*p* < 0.01, control vs. H_2_O_2_, *n* = 5), and decreased to −6.21 ± 0.45 pA/pF after the addition of Gö 6983 (*p* < 0.01, H_2_O_2_ vs. Gö 6983, *n* = 5). In the control experiments, the run-down of I_Ca,L_ was less than 5% [19].

### 2.4. PKC Mediates I_Na,L_ Augmentation in H_2_O_2_-Induced Afterdepolarizations

**Figure 5:**
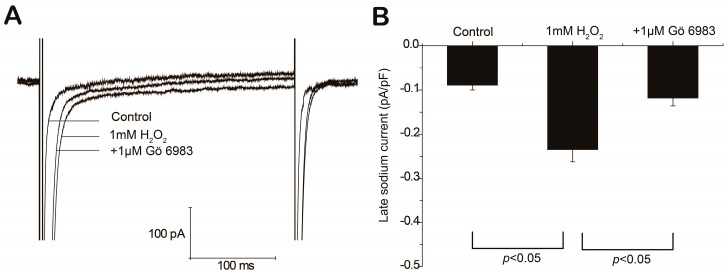
Effects of Gö 6983 on H_2_O_2_-induced late sodium current (I_Na,L_) elevation. Representative current-voltage traces (**A**) and summary data of I_Na,L_ at −30 mV under control condition, in the presence of 1 mM H_2_O_2_ alone or plus 1 μM Gö 6983 (**B**) are shown.

I_Na,L_ plays a key role in H_2_O_2_-induced afterdepolarizations [19,20,21]. I_Na,L_ curves averaged by five replicated depolarizing voltage steps from −120 to −30 mV of one representative cell are shown in Figure 5A. The amplitudes of I_Na,L_ were measured at 200 ms and are summarized in Figure 5B. Results showed that I_Na,L_ was elevated from −0.09 ± 0.01 to −0.23 ± 0.02 pA/pF (*p* < 0.01, control vs. H_2_O_2_, *n* = 7) and attenuated by 1 μM Gö 6983 to −0.11 ± 0.02 pA/pF (*p* < 0.01, H_2_O_2_ vs. Gö 6983, *n* = 7).

### 2.5. Less Involvement of I_to_ in PKC Signaling in H_2_O_2_-Induced Afterdepolarizations

**Figure 6:**
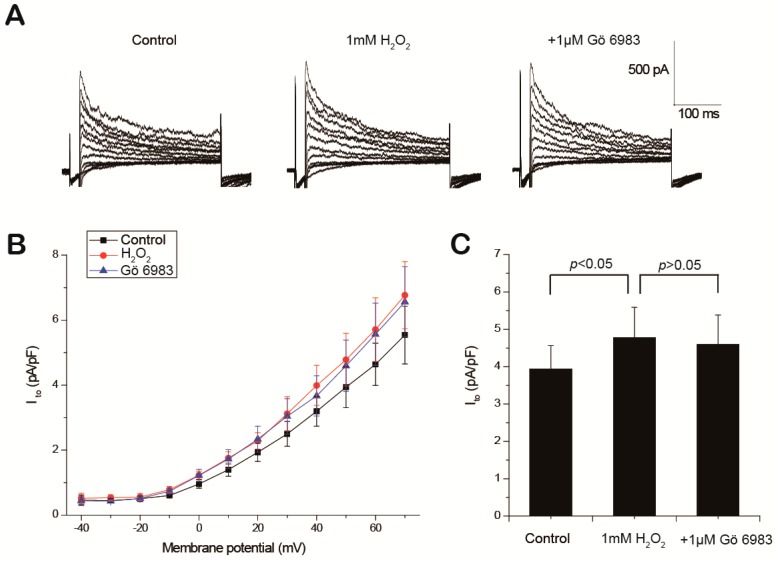
Effects of Gö 6983 on H_2_O_2_-induced transient outward potassium current (I_to_) elevation. Representative current–voltage traces (**A**), the averaged current-voltage curves of I_to_ (**B**) and summary histogram of I_to_ at +50 mV (**C**) under control condition, in the presence of 1 mM H_2_O_2_ alone or plus 1 μM Gö 6983 are shown.

The effects of H_2_O_2_ and the PKC inhibitor on I_to_ were also analyzed. The representative current-voltage traces of I_to_ are displayed in Figure 6A, and the averaged I–V curves (*n* = 6) are shown in Figure 6B. The peak amplitudes of I_to_ at +50 mV were measured and are summarized in Figure 6C. I_to_ was significantly elevated from 3.07 ± 0.62 to 3.88 ± 0.50 pA/pF after H_2_O_2_ perfusion (*p* < 0.05, control vs. H_2_O_2_, *n* = 6), and decreased to 3.58 ± 0.46 pA/pF after the addition of Gö 6983 (*p* > 0.05, H_2_O_2_ vs. Gö 6983, *n* = 6).

## 3. Discussion

The present study provided the following novel findings: (1) the specific classical PKC inhibitor Gö 6983 both prevented and reversed the emergence of H_2_O_2_-induced afterdepolarizations effectively, indicating PKC activation mediates oxidative stress-induced afterdepolarizations; (2) PKC inhibition by Gö 6983 attenuated H_2_O_2_-induced enhancement of I_Ca,L_ and I_Na,L_, but not I_to_.

PKC, a classical second messenger, is acknowledged to be involved in several pathological progressions including hypertrophy, fibrosis, inflammation, ischemic preconditioning and atherosclerosis. Thus, PKC serves as a potential therapeutic target for heart failure, hypertension and coronary heart diseases [10,22]. Previous studies have investigated the effect of PKC activation by endothelin-1 and a PKC agonist on arrhythmogenesis [16,23]. However, the relationship between PKC and arrhythmias in different pathological conditions, including oxidative stress, remains unclear.

Oxidative stress is involved in a variety of cardiovascular diseases, including heart failure and ischemic heart disease. Cardiac arrhythmias are prevalent in patients with these conditions. Afterdepolarization-mediated TAs play an important role in ROS-induced arrhythmias [24], but the underlying molecular mechanisms remain not fully elucidated. It is well known that classical PKC isozymes are activated by Ca^2+^ and diacylglycerol [25]. When inactive, the regulatory domain of PKC is bound to the catalytic domain, inhibiting the enzyme activity, and when PKC is activated, this autoinhibition is released [26]. It is noteworthy that, besides Ca^2+^ and diacylglycerol, mild oxidative stress can activate PKC by selectively oxidizing the regulatory domain [17]. These findings motivated us to explore the role of PKC signaling in oxidative stress-induced afterdepolarizations, and the present study provided direct evidence showing that PKC is indeed involved. In order to confirm that the results were not the side effects of PKC inhibitors, two widely used selective PKC inhibitors (Gö 6983 and BIM) were both tested, and the results demonstrated that it was the PKC inhibitory effect that affected H_2_O_2_-induced afterdepolarizations.

H_2_O_2_ may affect I_Ca,L_, I_Na,L_ and I_to_ via direct oxidative modification, intracellular signaling pathways or transcription and translation regulations [5]. Our results show that acute application of H_2_O_2_ is capable of enhancing these major membrane currents significantly. In our study, adding the PKC inhibitor along with H_2_O_2_ for several minutes effectively reversed the H_2_O_2_-induced alterations on I_Ca,L_ and I_Na,L_, suggesting the mechanistic role of PKC on the H_2_O_2_-induced intracellular signaling cascade. However, acute application of the PKC inhibitor failed to reverse the enhancement of I_to_, indicating a minor role of I_to_ on H_2_O_2_-induced effects.

When activated, PKC can phosphorylate downstream proteins including the L-type Ca^2+^ channel [27] and voltage-gated Na^+^ channel [28], resulting in I_Ca,L_ facilitation and I_Na,L_ augmentation. Previous studies have demonstrated that the elevation of not only I_Na,L_ but also I_Ca,L_ was involved in the ROS-induced afterdepolarization formation [3]; thus, elevation of I_Na,L_ can reduce the repolarization reserve, while activation of I_Ca,L_ can generate EAD upstroke. It is known that augmentation of I_Ca,L_ can increase Ca^2+^ influx and enhance cellular Ca^2+^ loading. In addition, APD prolongation can also enhance cellular Ca^2+^ loading, and further promotes PKC activation. In our study, the inhibition of PKC activation significantly reversed H_2_O_2_-induced enhancement of both I_Ca,L_ and I_Na,L_, these might result in reduced cellular Ca^2+^ loading by attenuating Ca^2+^ influx via I_Ca,L_ and shortened APD.

The results collectively suggested that PKC signaling plays a central role in oxidative stress-induced afterdepolarizations, although activation of other kinases such as protein kinase A and Ca/calmodulin-dependent protein kinase II (CaMKII) cannot be ruled out, and it is beyond the present study protocol. Previous studies have demonstrated that CaMKII activation played a role in H_2_O_2_-induced afterdepolarizations [3]. As PKC and CaMKII are both kinases that can be activated by the oxidative modification and elevated Ca^2+^ level, they may share similar ionic mechanisms in oxidative stress-induced afterdepolarizations. Another study demonstrated similar inhibitory effects on intracellular Ca^2+^-induced I_Na,L_ by both inhibitors of PKC and CaMKII [29]. This evidence together with our results indicates that PKC activation is essential in oxidative stress-induced afterdepolarizations.

It has already been demonstrated that ROS levels are elevated in a number of cardiovascular diseases and predispose the heart to arrhythmias. Free radical scavengers were supposed to be a potential therapy strategy for alleviating arrhythmias [30], but this proposal was found to be not effective due to some limitations [31]. Thus, downstream second messengers, such as PKC, may be potential novel therapeutic targets for arrhythmias. Regulators of PKC are already in clinical trials for different indications [32,33,34,35,36], and systemic delivery of activators and inhibitors of PKC has been proven to be well tolerated [32,33]. Future studies are warranted to test the safety and feasibility of PKC inhibitors to treat arrhythmias in experimental settings and in patients.

## 4. Materials and Methods

### 4.1. Solutions and Drugs

The standard Tyrode solution and Kraft-Bruhe (KB) solution were made with Milli-Q grade water. The standard Tyrode solution contained (in mM) 135 NaCl, 5.4 KCl, 0.33 NaH_2_PO_4_, 1.0 MgCl_2_, 10 glucose, 10 HEPES, 1.8 CaCl_2_ with pH adjusted to 7.4 with NaOH. The KB solution contained (in mM) 85 KOH, 50 K-glutamate, 30 KCl, 1.0 MgCl_2_, 20 taurine, 0.5 Ethylene glycol bis(2-aminoethyl ether) tetraacetic acid (EGTA), 10 glucose, 10 HEPES with pH adjusted to 7.4 with KOH. Chemicals and reagents were purchased from Sigma Aldrich (St. Louis, MO, USA) unless otherwise noted. BIM was purchased from Selleckchem (Houston, TX, USA). Nifedipine, Gö 6983 and BIM were dissolved in Dimethyl Sulfoxide (DMSO) as stock solution according to the manufacturer’s instructions before diluting into the superfusate solution at the final concentration. The final concentration of DMSO was less than 0.1%.

### 4.2. Cell Isolation

The use and care of animals conformed to the Guide for the Care and Use of Laboratory Animals of Shanghai, China and was approved by the Institutional Animal Care and Use Committee of Xinhua Hospital (Approval No.: XHEC-F-2016-009, Date: 22 March 2016). Hearts were removed from 23 adult New Zealand White rabbits (2 to 3 kg) anesthetized with intravenous pentobarbital, and were then perfused retrogradely in Langendorff apparatus at 37 °C with Ca^2+^-free Tyrode’s solution containing 1.4 mg/mL collagenase (Type II, Worthington, Lakewood, NJ, USA) and 0.1 mg/mL protease (type XIV, Sigma, St. Louis, MO, USA) for 15 to 30 min. After washing out the enzyme solution, the hearts were removed from the apparatus and swirled in a culture dish in KB solution. The Ca^2+^ concentration was gradually increased to 1.8 mM, and the cells were then kept at 4 °C until use.

### 4.3. Patch-Clamp Methods

All experiments were performed at 35 °C to 37 °C. Myocytes were perfused in standard Tyrode solution for 5 min and then patch-clamped using the whole-cell configuration. H_2_O_2_ (1 mM) and Gö 6983 (1 μM) were added to the bath superfusate. For AP recordings, patch pipettes (resistance 2 to 3 MΩ) were filled with pipette solution containing (in mM) 110 K-aspartate, 30 KCl, 5 NaCl, 0.1 EGTA,10 HEPES, 5 MgATP, 5 creatine phosphate, 0.05 cAMP, pH 7.2 with KOH. APs were elicited with 2 ms, 2–4 nA square pulses at basic cycle lengths of 6 s.

For I_Ca,L_ recording, the cells were superfused with a modified Tyrode’s solution in which KCl was replaced by CsCl, and the patch pipettes (resistance 1 to 2 MΩ) were filled with an internal solution containing (in mM): 110 Cs-Aspartate, 30 CsCl, 5 NaCl, 10 HEPES, 5 MgATP, 5 creatine phosphate, 0.1 EGTA, 0.05 cAMP, pH 7.2 with CsOH. I_Ca,L_ was recorded using a series of 200 ms steps from −40 to +50 mV with increments of 10 mV from a holding potential of −80 mV prior to a 100 ms prepulse to −40 mV.

To isolate I_Na,L_, the pipette solution contained (in mM) 110 Cs-aspartate, 30 CsCl, 10 HEPES, 0.5 EGTA, 0.2 Na_3_-GTP, 5 Na_2_-phosphocreatine, 5 MgATP, pH 7.2 with CsOH. The bath solution was the same as that used for I_Ca,L_. Nifedipine (30 μM) was added to the bath solution to block calcium currents. I_Na,L_ was elicited by 300 ms pulses from −120 to −30 mV, and the amplitude was measured at 200 ms.

For transient outward potassium current (I_to_) recording, the pipette and bath solutions were the same as those for AP recording. TTX (10 μM) and Cd^3+^ (0.3 mM) were added into the Tyrode’s solution to inhibit I_Na,L_ and I_Ca,L_. I_to_ was elicited at basic cycle lengths of 10 s by a series of 300 ms steps from −40 to +70 mV with increments of 10 mV from a holding potential of −70 mV prior to a prepulse to −40 mV.

### 4.4. Data Acquisition and Analysis

Voltage and current signals were measured with a MultiClamp 700B patch-clamp amplifier (Axon Instruments, Sunnyvale, CA, USA) controlled by a personal computer using a Digidata 1440A acquisition board driven by pCLAMP 10 software (Molecular Devices, Sunnyvale, CA, USA). Data are presented as means ± standard error of means unless indicated. Statistical significance was assessed using unpaired Student’s *t* tests, and *p* < 0.05 was considered significant.

## 5. Conclusions

PKC activation mediates oxidative stress-induced afterdepolarizations via enhancement of I_Ca,L_ and I_Na,L_, and PKC might be a potential antiarrhythmic therapeutic target.

## Supplementary Materials

Supplementary materials can be found at www.mdpi.com/1422-0067/18/4/688/s1.
