# Supplementary material for: Oxidative Stress-Induced Afterdepolarizations and Protein Kinase C Signaling
Source: Int J Mol Sci. 2017 Mar 30;18(4):688. doi: 10.3390/ijms18040688 (PMC5412274; doi:10.3390/ijms18040688)
Supplement: Supplementary file 1 [file ijms-18-00688-s001.pdf]

Supplementary Materials: Oxidative Stress-Induced Afterdepolarizations and Protein Kinase C Signaling

Yu-Dong Fei, Wei Li, Jian-Wen Hou, Kai Guo, Xiao-Meng Chen, Yi-He Chen, Qian Wang, Xiao-Lei Xu, Yue-Peng Wang and Yi-Gang Li

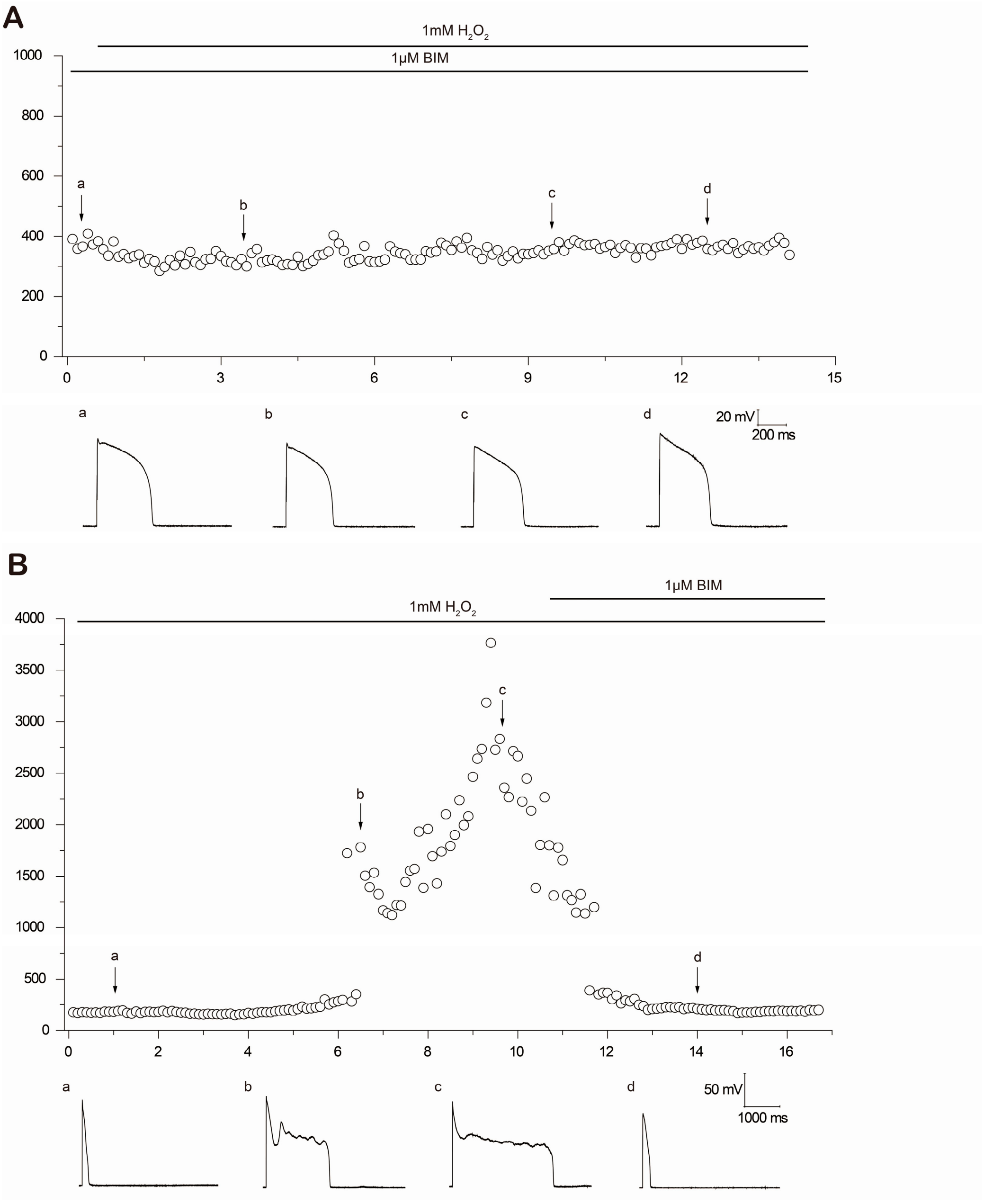

Figure S1. Effects of BIM on H_2O_2-induced early afterdepolarizations (EADs). (**A**). Time course of action potential durations (APD) 90 in a myocyte pretreated with BIM before exposure to 1 mM H_2O_2. Action potentials (APs) in the presence of BIM (a), after perfusion of H_2O_2 for 3 min (b), 9 min (c) and 12 min (d), are shown below. (**B**) Time course of APD 90 in a myocyte treated with BIM after EADs were induced by H_2O_2, as indicated by the horizontal bar. APs after perfusion with H_2O_2 for 1 min (a), 6 min (b) and 9 min (c) and after application of BIM (d) are shown below.
